# Trends of Obesity in 10-Years of Follow-up among Tehranian Children and Adolescents: Tehran Lipid and Glucose Study (TLGS)

**Published:** 2019-09

**Authors:** Farhad HOSSEINPANAH, Sara SERAHATI, Maryam BARZIN, Shayan ARYANNEZHAD, Maryam REZAIE, Majid VALIZADEH, Fereidoun AZIZI

**Affiliations:** 1. Obesity Research Center, Research Institute for Endocrine Sciences, Shahid Beheshti University of Medical Sciences, Tehran, Iran.; 2. Medical Toxicology and Drug Abuse Research Center (MTDRC), Birjand University of Medical Sciences, Birjand, Iran.; 3. Endocrine Research Center, Research Institute for Endocrine Sciences, Shahid Beheshti University of Medical Sciences, Tehran, Iran

**Keywords:** Obesity, Childhood, Trend, Iran

## Abstract

**Background:**

We aimed to investigate the trend of childhood obesity in Tehranian population during a median follow-up of 10 years.

**Methods:**

Within a prospective cohort study, using data collected from Tehran Lipid and Glucose Study (TLGS), 1406 participants, aged 3–11 yr were selected and monitored in 4 phases: phase I (1999–2001), phase II (2002–2005), phase III (2006–2008) and phase IV (2009–2011).

**Results:**

Total prevalence of obesity in children increased from 5.5% to 9.4% from phase I to IV. Performing GEE (Generalized Estimating Equation) analysis, relative risk of obesity was calculated, comparing each phase to its previous phase: phase II in reference to phase I (RR=1.06, CI=1.04–1.08), phase III in reference to phase II (RR=1.01, CI=1.00–1.03) and phase IV in reference to phase III (RR=0.96, CI=0.94–0.98). Between group difference was significant in all subgroups (age, gender, parental obesity) except parental education. Test of interaction for effect of time was insignificant in all subgroups except for the age group. For children younger than 7 yr old at phase I, trend of obesity throughout the study was higher compared to those with 7 yr of age and older at phase I.

**Conclusion:**

During a decade of follow-up, trend of obesity was rising in this Tehranian children in both genders, especially in younger children. Any preventive interventions for stopping this trend should focus on early stages of childhood.

## Introduction

Childhood obesity is considered to increase the risk of both premature mortality and adult cardiometabolic morbidities (1), such as diabetes, hypertension, coronary heart disease, non-alcoholic fatty liver disease, dyslipidemia and atherosclerosis (2). Globally, the high prevalence of overweight and obesity among children and adolescents has been alarming (3). In developed countries like the United States, an increasing rate in the prevalence of overweight among children between 1960s and 1999 has been reported (4), although no significant changes between 1999 and 2006 were observed (5). In the last decade the overall increasing trend of obesity in the United States had ceased (6). On the other hand, developing countries are facing an elevated prevalence of childhood obesity as well as a dramatic rise in its trend, as a consequence of rapid adoption of western lifestyle involving dietary changes and a decreasing trend in physical activity (7, 8).

Studies in Iran show an increase in the prevalence of overweight and obesity in the last decade, rising from 4.8% and 10.1% to 6% and 14% respectively (9, 10). A recent systematic review and meta-analysis also reported an escalating trend of excess weight among young Iranian children (11). However, these reports are based on cross-sectional survey data and have not tracked trends of obesity in a single population over time, and are therefore incapable of evaluating the effects of different risk factors, which predispose individuals to weight gain. Further longitudinal studies are essential to reveal these associations.

Previously, after 6.6 years of follow up, an increasing trend of childhood obesity was reported in first three phases of Tehran Lipid and Glucose Study (12). In this longitudinal study, using updated data available from phase IV of TLGS (2009–2011), we aimed to determine the prevalence and trend of obesity with 10 years of follow up among a population of Tehranian children, taking into account the effect of different risk factors on this trend.

## Materials and Methods

### Study participants and design

This longitudinal population-based study used data available from Tehran Lipid and Glucose Study (TLGS), a cohort study designed with the aim of determining the prevalence of non-communicable disease risk factors and its outcomes among the Tehranian urban population. For the baseline measurements, 15005 individuals, aged≥3 yr were selected by multistage cluster random sampling methods in phase I (1999–2001), and assessments were repeated in several subsequent phases at approximately 3.6 years intervals; phase II (2002–2005), phase III (2006–2008), and phase IV (2009–2011). Details of the study have been published elsewhere (13). For the current study, 1406 individuals aged 3–11 yr at the beginning of phase I, measured at least once again throughout phases II to IV were selected.

### Measurements and definitions

Trained interviewers used pretested questionnaires to collect information regarding demographics, smoking, and educational level. Smoking was categorized into a ‘yes’ group (participants who smoked cigarettes on a daily basis, occasionally or ex-smokers.) and a ‘no’ group (non-smokers). Educational level was defined in two levels, depending on either completion of at least 12 years of education (having a high school diploma or higher degree) or not.

Anthropometric examinations were conducted by trained personnel as well. Weight was measured with participants being minimally clothed without shoes using a digital electronic weighing scale (Seca 707, range 0.1–150 kg; Hanover, MD, USA) and was recorded to the nearest 100 g. Height was measured by a tape meter in standing position according to standard protocols. BMI was calculated as weight in kilograms divided by the height in square meters (kg/m^2^). Childhood obesity (2–19 yr) in each sex was defined as BMI-for-age>2SD based on standard tables of WHO (14), and parental obesity was defined as BMI ≥30.

### Statistical analysis

Continuous variables are expressed as means (SD), and categorical covariates are expressed as percentages. Tests of interactions were checked considering time trends and obesity for subgroups of age, father’s smoking status, and parental obesity and educational level. The response was dependent, the GEE model with auto-regressive working correlation structure, through log-link function with binomial errors, was used to investigate marginal means and *P*-values and estimate the relative risk(RR) of responses and 95% confidence intervals. All analyses were performed using IBM SPSS for Windows, ver. 20 (SPSS, Chicago, IL, USA); the significance level was set at *P*<0.05 (two-tailed).

## Results

For the current study, 1406 children (673 males) in phase I of TLGS who had at least one follow-up in phases II to IV comprised of the population sample; of these, number of participants in phases II, III and IV who had the information required were 964, 1111, and 1044 respectively. Characteristics of different subgroups of the whole study population in phases I to IV are shown in Table 1 and gender-based data are shown in Tables 2 and 3.

**Table 1:** Characteristics of different subgroups of study population in phases I to IV

***Variables***	***Phase I (1999–2001)***	***Phase II (2002–2005)***	***Phase III (2006–2008)***	***Phase IV (2009–2011)***
Age (yr) mean ±SD	7.7 ± 2.5	11.5 ±2.3	14.6 ±2.5	18.1±2.5
BMI(Kg/m^2^) mean ±SD				
Children	16.1 ±3.1	19.2 ±3.7	21.6 ±4.3	23.5 ±4.3
Fathers	26.1 ±3.8	26.9 ±3.3	27.3 ±3.6	27.6 ±3.6
Mothers	27.4±4.5	28.7 ±3.8	29.0 ±4.2	29.9 ±4.5
Childhood obesity n (%)				
Total	78 (5.5)	110 (11.7)	144 (13.0)	102 (9.4)
Boys	49 (7.3)	69 (14.6)	80 (14.9)	56 (10.9)
Girls	29 (3.9)	41 (9.0)	64 (11.2)	46 (8.1)
Parental obesity n (%)				
Fathers	171 (15.1)	173 (20.9)	218 (24.2)	215 (25.2)
Mothers	339 (25.4)	298 (33.7)	359 (35.6)	424 (42.8)
Smoking n (%)				
Fathers				
Smoker	538 (48.9)	385 (51.9)	415 (50.2)	417 (50.8)
Mothers				
Smoker	48 (3.7)	42 (4.5)	46 (4.12)	43 (4.6)
Educational level n (%)				
Fathers				
≤12	914 (82.6)	639 (80.5)	664 (78.5)	649 (78.5)
>12	193 (16.9)	154 (19.5)	197 (21.5)	191 (21.5)
Mothers				
≤12	1241 (92.5)	837 (90.9)	928 (89.8)	870 (88.3)
>12	101 (7.5)	77 (9.0)	102 (10.2)	121 (11.7)

BMI: Body Mass Index (kg/m^2^); Childhood obesity: BMI-for-age > 2SD in each sex, Parental obesity: BMI ≥30 kg/m^2^.

**Table 2:** Characteristics of different subgroups of study population in phases I to IV (Boys)

***Variables***	***Phase I (1999–2001)***	***Phase II (2002–2005)***	***Phase III (2006–2008)***	***Phase IV (2009–2011)***
Age (yr) mean ±SD	7.63 ±2.5	11.4±2.3	14.5 ±2.5	17.9±2.5
BMI(Kg/m^2^) mean ±SD				
Children	16.0 ±2.8	19.1 ±3.7	21.6 ±4.6	23.5±4.5
Fathers	26.3 ±3.8	27.0±3.4	27.5 ±3.6	27.7 ±3.6
Mothers	27.3 ±4.5	28.6 ±3.7	28.9 ±4.2	29.9 ±4.5
Obesity n (%)				
Children	49 (7.3)	69 (14.6)	80 (14.9)	56 (10.9)
Fathers	80 (14.6)	81 (21.2)	101 (23.7)	98 (23.8)
Mothers	167 (26.4)	145 (34.5)	175 (36.0)	194 (42.3)
Smoking n (%)				
Fathers				
Smoker	258 (47.8)	179 (49.8)	192 (47.9)	199 (49.3)
Mothers				
Smoker	23 (3.7)	18 (4.2)	17 (3.6)	20 (4.2)
Educational level n (%)				
Fathers				
≤12	450 (82.9)	304 (80.1)	331 (78.9)	314 (78.3)
>12	94 (17.0)	85 (19.9)	95 (21.0)	95 (21.7)
Mothers				
≤12	593 (92.5)	397 (90.9)	437 (89.6)	408 (87.6)
>12	48 (7.4)	40 (9.1)	52 (10.4)	56 (12.1)

BMI: Body Mass Index (kg/m^2^); Childhood obesity: BMI-for-age > 2SD in each sex, Parental obesity: BMI ≥30 kg/m^2^.

**Table 3:** Characteristics of different subgroups of study population in phases I to IV (Girls)

***Variables***	***Phase I (1999–2001)***	***Phase II (2002–2005)***	***Phase III (2006–2008)***	***Phase IV (2009–2011)***
Age (yr) mean ±SD	7.7 ±2.5	11.5 ±2.3	14.7 ±2.4	18.1 ±2.4
BMI(Kg/m^2^) mean ±SD				
Children	15.9 ±3.4	19.2 ±3.7	21.5 ±3.9	23.5 ±4.0
Fathers	25.9 ±3.7	26.7 ±3.1	27.0 ±3.5	27.4 ±3.5
Mothers	27.4 ±4.5	28.7 ±3.8	29.2 ±4.3	30.1 ±4.5
Obesity n (%)				
Children	29 (3.9)	41 (9.0)	64 (11.2)	46 (8.1)
Fathers	91 (15.3)	92 (20.4)	117 (24.3)	117 (26.3)
Mothers	172 (24.7)	153 (33.1)	184 (35.3)	230 (43.1)
Smoking n (%)				
Fathers				
Smoker	280 (50.3)	206 (54.3)	223 (52.6)	218 (52.5)
Mothers				
Smoker	25 (3.6)	24 (4.8)	29 (4.5)	23 (4.9)
Educational level n (%)				
Fathers				
≤12	464 (82.8)	335 (81.2)	333 (78.3)	335 (78.8)
>12	99 (17.0)	69 (18.8)	102 (21.7)	96 (21.2)
Mothers				
≤12	648 (92.4)	440 (91.1)	491 (89.9)	462 (88.8)
>12	53 (7.6)	37 (8.9)	50 (10.1)	65 (11.2)

BMI: Body Mass Index (kg/m^2^); Childhood obesity: BMI-for-age > 2SD in each sex, Parental obesity: BMI ≥30 kg/m^2^

Total prevalence of obesity increased from 5.5% to 9.4% from phase I to IV, being the highest at phase III (13.0%); the same pattern of increase was observed in both sexes, rising from 7.3% and 3.9% to 10.9% and 8.1% in boys and girls respectively, while it was the highest at phase III (14.9% and 11.2%). Other information regarding mean BMI, parental obesity, smoking status and parental educational level throughout the four phases are presented in Tables 1–3.

Performing GEE analysis, the relative risks of obesity in different subgroups of study population, each phase compared to its previous phase were calculated (Table 4). For the whole population, relative risk of obesity increased in phase II compared to phase I (RR=1.06, CI=1.04–1.08), remained steady in phase III, compared to phase II (RR=1.01, CI=1.00–1.03) and decreased in phase IV, compared to phase III (RR=0.96, CI=0.94–0.98). This pattern for relative risk of obesity was observed in all subgroups of study except for children in age group <7 (trend of obesity continued to rise until phase III and then decreased).

**Table 4:** Relative risks of obesity in different subgroups of study population, each phase compared to precede phase

	***Phase II (reference phase I)***		***Phase III (reference phase II)***		***Phase IV (reference phase III)***		***P-value of time interaction***
	***RR***	***CI***	***RR***	***CI***	***RR***	***CI***	
Total	1.06	1.04–1.08	1.01	1.03–1.00	0.96	0.98–0.94	
Sex							
Boys	1.08	1.11–1.04	1.00	1.03–0.98	0.96	0.98–0.93	0.526
Girls	1.06	1.08–1.03	1.02	1.04–1.00	0.97	0.99–0.95	
Age							
<7 years	1.08	1.12–1.04	1.06	1.09–1.02	0.99	1.02–0.95	<0.001
≥7 years	1.05	1.07–1.03	0.99	1.01–0.97	0.95	0.97–0.93	
Father’s obesity							
Obese	1.13	1.20–1.07	1.01	1.07–0.95	0.93	0.98–0.88	0.884
Non-obese	1.04	1.06–1.02	1.02	1.04–1.00	0.98	0.99–0.96	
Mother’s obesity							
Obese	1.10	1.15–1.06	1.02	1.05–0.98	0.92	0.96–0.89	0.638
Non-obese	1.04	1.06–1.02	1.01	1.03–1.00	0.98	1.00–96.0	
Father’s smoking status							
Smoker	1.05	1.08–1.02	1.02	1.05–0.98	0.97	1.00–0.94	0.560
Non-smoker	1.08	1.12–1.04	1.01	1.04–0.98	0.95	0.98–0.92	
Mother’s smoking status							
	NA	NA	NA	NA	NA	NA	-
Father’s education							
≤12	1.07	1.09–1.04	1.01	1.03–0.98	0.96	0.98–0.94	0.458
>12	1.04	1.50–0.72	1.04	1.59–0.68	0.95	0.99–0.92	
Mother’s education							
≤12	1.06	1.08–1.04	1.02	1.04–1.00	0.96	0.98–0.94	0.846
>12	1.08	1.16–1.01	1.00	1.06–0.94	0.98	1.03–0.93	

RR: Relative risk; Parental obesity : BMI ≥30 Kg/m^2^; Childhood obesity: BMI-for-age > 2SD in each sex; NA: data not applicable

**Fig. 1:**
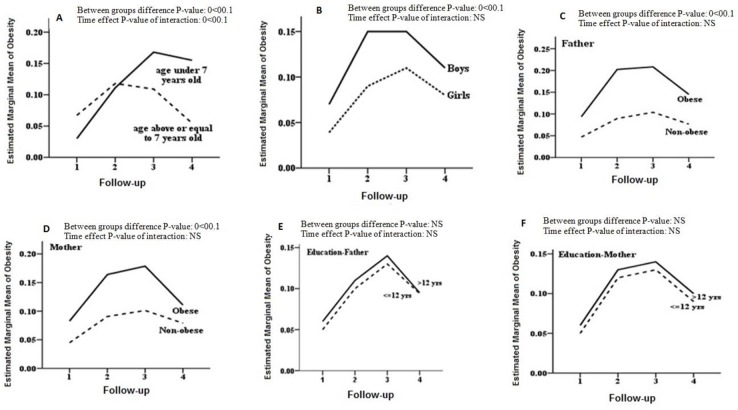
Trend of obesity in different subgroups from 1999 to 2011; A. Stratified by age group; B. Stratified by gender; C. Stratified by Father’s obesity; D. Stratified by mother’s obesity; E. Stratified by father’s educational level; F. Stratified by mother’s educational level. // NS: Not significant

During 4 phases of study, between group difference was statistically significant (*P*-value <0.01) in all subgroups (Fig. 1.A–D) except for education of parents (Fig. 1.E–F). In subgroup analysis, test of interaction for effect of time was insignificant in all subgroups (sex, parental obesity, parental educational level) except for the age group (*P*-value <0.001). Children, aged<7 yr, had an increasing risk of obesity in phases II (RR=1.08, CI=1.04–1.12) and phase III (RR=1.06, CI=1.02–1.08) compared to phases I and II respectively, but relative risk of obesity in phase IV, compared to phase III was 0.99 (CI=0.95–1.02).

For those aged≥7 yr, relative risk of obesity at phase II compared to phase I was (RR=1.05, CI=1.03–1.07), but started to decrease in phases III compared to II (RR=0.99, CI=0.97–1.01) and phase IV compared to III (RR=0.95, CI=0.93–0.97) (Table 4, Fig. 1.A).

## Discussion

In this study, after 10 years of follow-up in urban Tehranian children, we have found an overall increasing trend of obesity, higher in <7 yr old children, although a slight decrease in the trend was observed in the final years of follow-up. Boys and children with obese parents had higher prevalence of obesity in all phases compared to girls and those with non-obese parents, their trend of obesity followed the same pattern throughout the study.

Even though childhood obesity is recognized as a major health issue in developing countries, most studies addressing it are cross-sectional based and are hence impractical for analysis of the problem over time. To the best of our knowledge, this is the first report of childhood obesity trend and its risk factors in the MENA (Middle East and North Africa) region within the framework of a longitudinal cohort study. Previously, using data from 144 countries, it was reported that childhood overweight and obesity had increased dramatically since 1990 worldwide (15). It was shown while the prevalence of childhood obesity is higher in developed countries compared to developing ones (11.7% vs. 6.1%), the relative percentage change was higher in the developing (an increase of 65% between 1990 and 2010) compared to developed countries (an increase of 48% between 1990 and 2010), findings in agreement with ours, as a developing country (childhood obesity prevalence of 5.5% in 1999 and 9.4% in 2011). However, nationally representative cross-sectional surveys were used as data source whereas our findings were provided in a longitudinal cohort study of a similar population over time, which makes it more justifiable.

Pattern in the trend of childhood obesity has reached a plateau in developed countries of Europe and in the United States (15–19). In the United States, National Health and Nutrition Examination Survey (NHANES) results, based on cross-sectional analyses of a representative sample of children and adolescents indicated that the prevalence of obesity had an increasing trend from 1971–.1974 to 1988–1994 in 6–11 and 12–19 yr old youth (4.0% and 6.1% to 11.3% and 10.5% respectively) a trend that continued until 1999–2000 (15.3% and 15.5%) (4). Overall youth obesity was reported to be 16.3% in 2003–2006 (5), and 17.0 % in 2013–2014 (17%) (20). In the latest report including the data of 1988–1994 to 2013–2014, for the 2–5 yr old children, the prevalence of obesity increased until 2003–2004 and then decreased; for the 6–11 yr increased until 2007–2008 and then leveled off, and for the adolescents aged 9–12 is still increasing (21). In contrast to our findings, in USA the overall prevalence of obesity was similar in girls and boys among youth, aged 2–19 yr. Such contradictions with our findings can be explained by differences in our sample populations, such as use of different age groups, different socio-economic background and diversity of ethnicity in the NHANES study. Overall, developing and developed countries show a similar pattern of increasing prevalence of obesity, but are currently at different stages, as our results share similarities with those reported by NHANES for United States from the 1970s to 1990s.

As a developing country, our results are also somehow in contradiction with recent reports of Europe and United States as the nature of their population differs at so many levels (socio-economic status, use of preventative strategies and etc.) with ours. Therefore, it seems more practical to consider studies carried out in developing countries. In line with our findings, using nationally representative data, prevalence of overweight among older children increased in Brazil (from 1975 to 1997) and China (from 1991 to 1997) significantly, as the burden of nutritional problems has shifted from energy imbalance deficiency to excess weight gain in those countries (22). Moreover, these findings are confirmed by more recent studies reporting a rising trend of childhood obesity in China (23) and India (24).

To address the prevalence and trends of childhood obesity and overweight in Iran, a systematic review and meta-analysis was published in 2014 (11). Reporting an increasing trend in the prevalence of childhood obesity, it was similar to that reported by us, but not as sharp as ours (7.25% in 2005–2010 compared to 5.13% in 1990–1995); there are some possible explanations for this difference in the degree of trend escalation; data were used from 109 studies conducted in all parts of Iran, including rural regions, their results cannot be representative of the urban population of Tehran as was the case in our study, and the CDC definition criteria used to define childhood obesity, whereas we used WHO standard tables.

In the current study we have addressed some important factors for childhood obesity. First, our results demonstrated that younger children (those who were under 7 yr old at baseline) were more prone to gain excess weight compared to older ones, a finding in agreement with previous reports which indicated that higher prevalence of obesity are in children more likely to occur at younger ages (25), demonstrating that onset of obesity begins early in childhood and to prevent the ascending trend of obesity, preventive strategies better focus on children earlier, probably before the age of 7. In fact, by considering the role of early ages in childhood obesity, we can explain the unexpected decreased relative risk of obesity in phase IV, compared to phase III, as participants have grown older during the cohort and were no longer ‘young children’ after 10 years of follow up. Another reason for the latter decreased risk might be the educations given to the participants of program, by rising their knowledge about lifestyle improvements. Moreover, we also found that another important risk factor for childhood obesity is having obese parents, a finding reported consistently, showed parental obesity predicts future weight gain among children (26, 27).

We are aware that our research may have some limitations. First, no data regarding physical activity, dietary habits, weight at birth and economic status of participants was documented. Second, our results cannot be extrapolated to the country’s population, as our subjects were representative of only Tehranian. And third, because our sample size was not large enough we were unable to divide our participants into smaller age subgroups allowed us to better estimate the role of age in our results.

However, this study has some noteworthy strengths. Being the first research in the MENA region to investigate the childhood obesity trend in a longitudinal cohort study design, with 10 years of follow up, is our main strength. Regarding anthropometric data, we used trained personnel to collect data, rather than using self-reported measures which would potentially underestimate obesity prevalence (28). We also used GEE analysis for statistical modeling, which is an advanced method for handling missing data.

## Conclusion

There is a rising trend of obesity among Tehranian children, especially at younger ages. We suggest that health policy makers prioritize early stages of childhood, perhaps pre-school ages to implement interventions strategies for stabilization of this ascending trend. Further studies are definitely needed to monitor the childhood obesity trend in Iran. Addressing the role of predisposing risk factors in this trend also needs to be given more attention.

## Ethical considerations

Ethical issues (Including plagiarism, informed consent, misconduct, data fabrication and/or falsification, double publication and/or submission, redundancy, etc.) have been completely observed by the authors.
